# Nutrient production from Korean poultry and loading estimations for cropland

**DOI:** 10.1186/s40781-018-0160-1

**Published:** 2018-02-19

**Authors:** Seunggun Won, Naveed Ahmed, Byung-Gu You, Soomin Shim, Seung-Su Kim, Changsix Ra

**Affiliations:** 1 Department of Animal Resources, College of Life & Environmental Science, Daegu University, Gyeongsan, 38453 South Korea; 2 Division of Animal Resource Science, College of Animal Life Science, Kangwon National University, Chuncheon, 24341 South Korea

**Keywords:** Poultry manure, Nutrient loading coefficient, Volatile solids, Total nitrogen, Total phosphorus

## Abstract

**Background:**

Poultry breeding has increased by 306% in Korea, inevitably increasing the production of manure which may contribute to environmental pollution. The nutrients (NP) in the manure are essential for crop cultivation and soil fertility when applied as compost. Excess nutrients from manure can be accumulated on the land and can lead to eutrophication. Therefore, a nutrient load on the finite land should be calculated.

**Methods:**

This study calculates the nutrient production from Korean poultry by investigating 11 broiler and 16 laying hen farms. The broiler manure was composted using deep litter composting while for layer deep litter composting, drying, and simple static pile were in practice. The effect of weight reduction and storing period during composting was checked. Three weight reduction cases of compost were constructed to calculate nutrient loading coefficients (NLCs) using data from; i) farm investigation, ii) theoretical P changes (ΔP = 0), and iii) dry basis.

**Results:**

During farm investigation of broiler and layer with deep litter composting, there was a 68 and 21% N loss whereas 77 and 33% P loss was found, respectively. In case of layer composting, a loss of 10-56% N and a 52% P loss was observed. Drying manure increased the P concentrations therefore NLCs calculated using dry basis that showed quite higher reductions (67% N; 53% P). Nutrient loss from farm investigation was much higher than reported by Korean Ministry of Environment (ME).

**Conclusions:**

Nutrients in manure are decreased when undergo storing or composting process due to microbial action, drying, and leaching. The nutrient load applied to soil is less than the fresh manure, hence the livestock manure management and conservation of environment would be facilitated.

## Background

Global poultry breeding have gone under enormous industrialization since late 1950s that resulted in the generation of massive amounts of nutrients (N & P) concentrated in farms [1]. Traditionally, poultry litter is used for land amendment purposes and is spread on the soil as fertilizer. Poultry litter is composed of manure, bulking material, feed, bird feathers etc. and has a very high nutritional value [2]. Excessive use of poultry manure in cropping may result in over enrichment of the soil and may contribute to the nitrate pollution in the water bodies [3] and cause eutrophication [4]. The presence of very high nitrate concentrations in the drinking water may contribute to greenhouse gas emission, methamoglobinaemia, respiratory illnesses, and fetal abortions in livestock [5, 6].

In Korea, the main categories of livestock reported by the Ministry of Environment (ME) in 2012 are dairy cattle (0.47 M), beef cattle (3.16 M), swine (10.6 M), and chicken and ducks (205.9 M) [7]. The proportion of manure production related to the livestock type was 39% produced by swine, 34% produced by beef cattle, 13% produced by dairy cattle, and 14% produced by chicken. The total amount of manure produced in Korea was 45,306 kt/year in 2012. Prior to land application, manure produced in Korea is stored and composted by livestock farm owners. Despite having comparatively higher number of heads, chicken produce only 14% of the total manure produced by livestock in Korea.

Nutrient losses from livestock manure management systems occur in decreasing fashion based on reactivity, speciation, solubility, and fugacity of the nutrients in the following fashion: C, N, > > S > K, Na, Cl, B, > P and other minerals. The C and N show dual mobility in water soluble compounds and gases that cause very quick and complex losses compared to other minerals. Carbon losses are mainly in the gaseous form (CO_2_ and CH_4_), in dissolved forms (HCO_3_, DOC), and runoffs [8]. During composting, nitrogen is lost through NH_3_ volatilization, denitrification, and leaching; as a result there is a loss of nutrients to oxidation and microbial degradation depending on composting method and duration. Substantial discharges of NH_3_, CH_4_, and N_2_O occur during the composting process [9]. The N losses of raw material during poultry composting was seen as 38-46% of initial N as NH_3_ emission [10] and 0.2-6.0% N_2_O emissions [11, 12]. During storage period, the N losses are 20-40%.

The nutrient loadings on land in Korea were calculated using standard unit for manure production by ME or foreign agencies e.g. The Organization for Economic Co-operation and Development (OECD) and were excessively reported due to nutrient production calculations based on number of livestock heads and the excretion rates. The nutrient values were reported as if manure was directly applied to the land after production. Contrary to the usually reported nutrient loadings most of the manure (88.7%) generated in Korea is usually composted and a small liquid portion (9.1%) is released into the environment after treatment. The nutrient losses during composting or drying period were not considered when nutrient production was calculated by ME or OECD. Therefore, the purposes of this study were; (i) to evaluate composting methods used in poultry manure management, (ii) to calculate weight loss and nutrient loss during composting period, (iii) to estimate practical nutrient loadings on agricultural land from poultry manure after composting period and to calculate the coefficients for organics, nitrogen, and phosphorus through the composting and storage period.

## Methods

### The field survey and sampling methods:

Raw manure and compost samples were collected for over a period of 6 months (March to August 2014). A total of 27 poultry farms were investigated including 11 broiler farms at Gapyeong County, Hongcheon, Chuncheon, and 16 laying farms at Hwacheon area. To obtain basic information and manure management practices a survey of individual farms was carried out using questionnaire. Information for farm address, number of heads, excretion type, barn area, annual manure production and treatment methods, bulking material and annual composting production and methods etc., were collected. Fresh manure and compost were sampled on site in a 20-L bucket and later analyzed.

### Analytical methods

All samples were stored at − 4 °C until analyzed. Pre-treatment of samples was done by mixing with H_2_SO_4_ and heating in block digester (BD40, LaChat, USA) at 320 °C, prior to the measurement of total nitrogen (TN) and total phosphorus (TP) using auto water analyzer (QuikChem 8500, LaChat, USA) for poultry manure and compost samples. Total solids (TS) and water content were calculated by drying samples at 105 °C for 24 h and then volatile solids (VS) were measured by heating a pre-dried sample in a muffle furnace at 550 °C for 2 h. All analyses were done according to the standard methods [13]. The bulk density (weight per unit volume) of the samples was done on site using 18 L of bucket according to Cooperband method [14].

### Calculation of nutrient loading amount and nutrient loading coefficient:

The nutrient loading is the amount of nutrients from poultry manure released into the environment, particularly soil and water resources. Typically, livestock manure is composted or stored and converted into organic fertilizer prior to land spreading. Through this process, oxidation and volatilization of organic matter and nitrogen occurs and causes loss of nutrients depending on the composting method and period. Therefore, nutrient loss ratio is the reciprocal of nutrient loading coefficients (NLCs) which helps in calculating nutrient variations in the form of a factor, as manure is composted [7]. All the calculations for NLCs were carried out using following equations;

1 $$ \mathrm{Nutrient}\ \mathrm{loading}\ \mathrm{coefficient}\ \left(\mathrm{NLC}\right)=\frac{\mathrm{Total}\ \mathrm{nutrients}\ \mathrm{in}\ \mathrm{compost}\ {\left(\mathrm{VS},\mathrm{N},\mathrm{P}\right)}^{(a)}}{\mathrm{Total}\ \mathrm{nutrients}\ \mathrm{in}\ \mathrm{manure}\ {\left(\mathrm{VS},\mathrm{N},\mathrm{P}\right)}^{(b)}} $$

2 $$ (a)\ \mathrm{Total}\ \mathrm{Nutrients}\ \mathrm{in}\ \mathrm{Compost}=\mathrm{Total}\ \mathrm{amount}\ \mathrm{of}\ \mathrm{compost}\ \mathrm{produced}\ {\left(\frac{\mathrm{kg}}{\mathrm{d}}\right)}^{(e)}\times \mathrm{Concentration}\ \mathrm{of}\ \mathrm{nutrients}\ \mathrm{in}\ \mathrm{compost}\ \left(\mathrm{VS},\mathrm{N},\mathrm{P}\right)\left(\frac{g}{\mathrm{kg}}\right) $$

3 $$ (b)\ \mathrm{Total}\ \mathrm{Nutrients}\ \mathrm{in}\ \mathrm{Manure}=\mathrm{Total}\ \mathrm{amount}\ \mathrm{of}\ \mathrm{manure}\ \mathrm{produced}\ {\left(\frac{\mathrm{kg}}{\mathrm{d}}\right)}^{(c)}\times \mathrm{Concentration}\ \mathrm{of}\ \mathrm{nutrients}\ \mathrm{in}\ \mathrm{manure}\ \left(\mathrm{VS},\mathrm{N},\mathrm{P}\right){\left(\frac{g}{\mathrm{kg}}\right)}^{(d)} $$

4 $$ (c)\ \mathrm{Quantity}\ \mathrm{of}\ \mathrm{manure}\ \mathrm{produced}\ \left(\frac{\mathrm{kg}}{\mathrm{d}}\right)=\mathrm{Questionnaire}=\mathrm{ME}\ \mathrm{value}\ \left(\frac{\mathrm{kg}}{1000\ \mathrm{head}}\cdotp \mathrm{d}\right)\times \mathrm{The}\ \mathrm{number}\ \mathrm{of}\ \mathrm{heads} $$

5 $$ (d)\ \mathrm{Nutrient}\ \mathrm{concentration}\ \mathrm{in}\ \mathrm{manure}\ \left(\mathrm{VS},\mathrm{N},\mathrm{P}\right)\ \left(\frac{g}{\mathrm{kg}}\right)=\mathrm{Values}\ \mathrm{from}\ \mathrm{sample}=\mathrm{Values}\ \mathrm{from}\ \mathrm{ME} $$

6 $$ (e)\ \mathrm{Quantity}\ \mathrm{of}\ \mathrm{compost}\ \mathrm{produced}\left(\frac{\mathrm{kg}}{\mathrm{d}}\right)=\mathrm{Mixture}\ \mathrm{of}\ \mathrm{manure}\ \mathrm{and}\ \mathrm{bulking}\ \mathrm{material}\ {\left(\frac{\mathrm{kg}}{\mathrm{d}}\right)}^{(f)}\times \left(1-\mathrm{weight}\ \mathrm{loss}\ \mathrm{rate}\ {\left(\%\right)}^{(g)}\right) $$

7 $$ (f)\ \mathrm{Mixture}\ \mathrm{of}\ \mathrm{manure}\ \mathrm{and}\ \mathrm{bulking}\ \mathrm{material}\ \left(\mathrm{MMB}\right)\left(\frac{\mathrm{kg}}{\mathrm{d}}\right)=\mathrm{Manure}\ \mathrm{from}\ \mathrm{questionnaire}\ {\left(\frac{\mathrm{kg}}{\mathrm{d}}\right)}^{(c)}+\mathrm{Bulking}\ \mathrm{material}\ \left(\frac{\mathrm{kg}}{\mathrm{d}}\right)=\mathrm{Manure}\ \mathrm{from}\ \mathrm{ME}\ \left(\frac{\mathrm{kg}}{\mathrm{d}}\right)+\mathrm{Bulking}\ \mathrm{material}\ \left(\frac{\mathrm{kg}}{\mathrm{d}}\right) $$

8 $$ (g)\ \mathrm{Rate}\ \mathrm{of}\ \mathrm{weight}\ \mathrm{loss}\ \left(\%\right)=1-\frac{\mathrm{Quantity}\ \mathrm{of}\ \mathrm{compost}\ \mathrm{produced}\ \left(\frac{\mathrm{kg}}{\mathrm{d}}\right)}{\mathrm{Mixture}\ \mathrm{of}\ \mathrm{manure}\ \mathrm{and}\ \mathrm{bulking}\ \mathrm{material}\ \left(\frac{\mathrm{kg}}{\mathrm{d}}\right)}\  in\ \mathrm{Questionnaire}\kern1.5em $$

9 $$ {\displaystyle \begin{array}{l}\begin{array}{l}\mathrm{Weight}\ \mathrm{loss}\ \mathrm{rate}\ \left(\%\right)=\mathrm{Theoretical}\ \mathrm{P}\ \mathrm{content}\ \mathrm{changes}=0=\Delta \mathrm{P}=0=\kern0.5em 1\kern0.5em -\kern0.5em \frac{\left(\frac{\mathrm{Total}\ \mathrm{concentration}\ \mathrm{of}\ \mathrm{P}\ \mathrm{in}\ \mathrm{manure}\ \left(\frac{\mathrm{kg}}{\mathrm{d}}\right)}{\mathrm{Total}\ \mathrm{concentration}\ \mathrm{of}\ \mathrm{P}\ \mathrm{in}\ \mathrm{compost}\ \left(\mathrm{kg}\right)}\right)}{\mathrm{Mixture}\ \mathrm{of}\ \mathrm{manure}\ \mathrm{and}\ \mathrm{bulking}\ \mathrm{material}\ \left(\frac{\mathrm{kg}}{\mathrm{d}}\right)}\kern0.5em in\kern0.5em \mathrm{Deep}\kern0.5em \mathrm{litter}\kern0.5em \mathrm{compost}\kern0.5em \left(\mathrm{broiler}\kern0.5em \mathrm{and}\kern0.5em \mathrm{layer}\right)\\ {}\end{array}\\ {}=\kern0.5em 1\kern0.5em -\kern0.5em \frac{\mathrm{Total}\ \mathrm{concentration}\ \mathrm{of}\ \mathrm{P}\ \mathrm{in}\ \mathrm{MMB}\ \mathrm{before}\ \mathrm{compost}\mathrm{in}\mathrm{g}\left(\frac{\mathrm{mg}}{\mathrm{kg}}\right)}{\mathrm{Total}\ \mathrm{concentration}\ \mathrm{of}\ \mathrm{P}\ \mathrm{in}\ \mathrm{compost}\ \left(\frac{\mathrm{mg}}{\mathrm{kg}}\right)}\kern0.5em in\ \mathrm{drying}\ \mathrm{and}\ \mathrm{simple}\ \mathrm{static}\ \mathrm{pile}\ \mathrm{compost}\end{array}} $$

10 $$ \mathrm{Weight}\ \mathrm{loss}\ \mathrm{rate}\ \left(\%\right)=\mathrm{Dry}\ \mathrm{basis}\ \mathrm{of}\ \mathrm{dry}\ \mathrm{compost}=1-\frac{100-\mathrm{Moisture}\ \mathrm{content}\ \mathrm{of}\ \mathrm{manure}\ \left(\%\right)}{100-\mathrm{Moisture}\ \mathrm{content}\ \mathrm{of}\ \mathrm{compost}\ \left(\%\right)}\  in\ \mathrm{dry}\ \mathrm{basis}\ \mathrm{of}\ \mathrm{dry}\ \mathrm{compost} $$

### Scenario construction for NLC calculation

Nutrient concentrations were calculated for the total amount of manure and compost nutrients. The statement of farmers through questionnaire is not precise, so, to get accurate picture several combinations of scenarios were considered.

- The total amount of nutrients in manure:

There were total three cases constructed for total amount of nutrients produced from manure using Eq. 4 and Eq. 5. Case I was calculated by multiplying the total amount of manure produced in farm investigation by the concentration of nutrients present in farm samples. The volume (m^3^) of manure as claimed by farmers was first converted into weight (kg) through bulk density of sample (kg/m^3^) and then was divided by number of days (kg/d). Case II was calculated by considering the manure production from reports of (ME) and then multiplied by the concentration of nutrients in farm samples. For Case III both the manure production and concentrations of the nutrients were taken from ME reports. For broiler and layer deep litter method, only case II was used for manure production calculations because it was not possible to investigate on farm due to the immediate mixing of manure with litter whereas for layer-drying and layer simple static pile composting all three cases of manure production were considered.

b. The total amount of nutrients in compost:

The quantity of compost was calculated using Eq. 6 and Eq. 7. The scenarios for total amount of nutrients present in compost based on weight reduction rate were total of three cases. All three scenarios are denoted by capital alphabets (A, B, and C). Scenario A was the amount of weight reduction as observed in the farm compost in this study (Eq. 8). The compost volume (m^3^) was converted to weight (kg) through bulk density of compost (kg/m^3^) and then it was divided by number of days (kg/d). Scenario B was the comparison of phosphorus concentrations in manure and compost Eq. (9), assuming the theoretical P losses during the composting process, concentration in the manure, and to estimate the production of compost. It means that the total amount of Phosphorus in the composting process is theoretically unchanged. Phosphorus losses during the composting process are theoretically zero (ΔP = 0) but it is reported as it was lost, actually loss occurs due to leakage caused by leachate or run-off [15], discharge of nutrients due to leachate or run-off should be included in the nutrient load due to soil movement and water environment (Eq. 9). Scenario C was the weight reduction rate based on dry basis of the manure and compost by ignoring moisture content (Eq. 10).

## Results and discussions

### Farm investigation

Korean poultry farms can roughly be divided into broiler and layer hen farms. The basic information collected from investigated farms is summarized in the Table 1. A total of 27 poultry farms were investigated, among which 11 farms were raising broilers with the farm size averaging 73,363 ± 30,303 heads which can be converted to 115,000 ± 92,717 heads/farm in a year based on the number of litter exchange per year. Broilers were raised on flat surface for 35 days for 4.6 ± 1.3 times in a year averaging 162 ± 45 days per year. Manure was immediately mixed with the litter as excreted and blended due to the movement of the broiler, leading to composting. All farmers were using rice hull as broiler litter, water was added in manure litter mixture after raising period and the compost was taken out. The use of litter amount varied greatly as 10.4 ± 13 g/head·d, that depended on whether litter was reused or not for each farm. Some farmers did not reuse the litter while some dried and reused litter approximately four times by mixing with new litter. Choi et al. (2011) reported that reuse of litter could reduce farmers’ burden of litter purchase, and did not affect productivity and viral pathogen contamination [16], but there was a risk of increased bacterial pathogen contamination and ammonia production [17–20]. The broiler manure was mixed with the litter immediately after excretion and could not be sampled. Therefore, the manure excretion of broiler chicken was reported to be 85.5 g/head·day according to the ME (Calculated per 1000 heads; All the units of excretion (g/head.day) from chicken are based on 1000 heads in the text.). The daily amount of compost produced per head was 14.8 ± 12 g/head·d, which showed a large variation according to the amount of litter.

**Table 1:** Basic information obtained from investigated farms through questionnaire

Contents	Broiler	Layer
The number of farms	11	16
Breeding period (days)	35	365
Farm size (The number of poultry at a time)	73,363 ± 30,303	59,813 ± 48,410
The number of heads per farm	115,000 ± 92717^a^	–
Annual litter reuse (times/year)	1.5 ± 0.7	–
Bulking material used	Rice hull	Rice hull and sawdust or no bulking material
Daily litter usage per head (g/head.day)	10.4 ± 13	17.3 ± 17.6
Daily manure production (kg/day)	9833 ± 7927	7223 ± 6057
Daily manure production per head (g/head.day)	85.5^b^	116 ± 16.2
Daily compost production (kg/day)	1092 ± 942	2405 ± 3057
Daily compost produced per head (g/head.day)	14.8 ± 12	35.9 ± 21

^a^calculated from the number of the litter exchange

^b^Values adopted from ME (Calculated per 1000 heads)

The survey of 16 layer hen farms was done. The number of heads averaging 59,813 ± 48,410 and ranged from 6500 to 150,000, bred throughout the year. The laying hens’ manure management is largely divided into two types, either litter is spread on the flat surface there manure is excreted directly on litter or in caged breeding where manure is accumulated under the cage and later on it is transferred to composting facility for composting. First method of litter composting is similar to the broiler composting method while caged composting method is divided into dry composting and simple static pile composting. Dry composting uses a method of in-house drying or machine drying. In simple static pile composting the manure is mixed with bulking material. A total of 3 farms were using littering compost, 4 farms were using dry composting, and 9 farms were using simple static pile composting. The excretion of manure by laying hens was 116 ± 16 g/head.day, which was similar to that of 127 g/head.day, reported by ME. Either rice hull or sawdust was used as bulking material averaging 17.3 ± 17.6 g/head·day per laying hen while in dry composting no bulking material was used. The average daily amount of compost produced was 35.9 ± 21 g/head·day.

### Nutrient production in poultry manure and compost:

Since, broiler manure could not be sampled because manure was mixed with litter immediately after excretion, hence, nutrient values of 12.9 g N/kg and 4.7 g P/kg of broiler N and P reported by the ME were obtained. Production of nutrients in manure and compost can be observed in Table 2. The mean VS, N, and P concentrations of compost were 157.0 g VS/kg, 29.8 g N/kg, and 8.7 g P/kg.

**Table 2:** Nutrient production and concentrations in manure and compost of broiler and laying hens

Contents			Daily production per head (g/head·d)	Moisture content (%)	Bulk density (kg/m^3^)	Nutrients	Concentrations (g/kg)
Broiler	Manure		85.5^a^	–	–	VS	— ^b^
						N	12.9^a^
						P	4.7^a^
	Compost		14.8 ± 11.5	33.1 ± 9.3	257.3 ± 75.8	VS	157.0 ± 55.8
						N	29.8 ± 22.9
						P	8.7 ± 5.6
Layer	Manure		116 ± 16.2	74.0 ± 2.3	998.0 ± 68.8	VS	175.2 ± 30.1
						N	13.3 ± 2.7
						P	3.9 ± 1.3
	Compost	Deep litter	52.8 ± 44.8	51.1 ± 11.8	462.7 ± 116.1	VS	333.3 ± 36.0
						N	19.5 ± 8.3
						P	5.5 ± 0.7
		Drying	35.4 ± 15.1	24.4 ± 2.2	431.7 ± 199.0	VS	642.5 ± 61.6
						N	29.4 ± 12.9
						P	13.8 ± 3.7
		Simple static pile	30.4 ± 7.6	39.1 ± 22.7	338.3 ± 90.3	VS	387.0 ± 148.3
						N	21.2 ± 8.1
						P	6.6 ± 1.8
		Average	35.9 ± 20.5	–	385 ± 131	VS	441 ± 166
						N	23 ± 9.6
						P	8.2 ± 4.0

^a^Values adopted from ME (Calculated per 1000 heads)

^b^Organic component of the ME is not to be compared referred to as BOD

The VS, TN, and TP contents of layer hens’ manure was 175.2 ± 30.1, 13.3 ± 2.7, and 3.9 ± 1.3 g/kg respectively. TN and TP both had higher values than reported by ME (8.8 g N/kg and 3.2 g P/kg). The average nutrients obtained in layer compost were 441 ± 166 g VS/kg, 23 g N/kg, and 8.2 g P/kg. Whereas, in case of laying hens’ deep litter compost the concentrations were 333.3 ± 36.0, 19.5 ± 8.3, and 5.5 ± 0.7 g/kg for VS, TN, and TP respectively. For the dry compost, the concentrations were 642.5 ± 61.6, 29.5 ± 12.9, 13.8 ± 3.7 g/kg for VS, TN, and TP respectively, while for simple static pile compost the concentrations were 387.0 ± 148.3, 21.2 ± 8.1, and 6.6 ± 1.8 g/kg while for the dry compost, it was 642.5 ± 61.6, 29.5 ± 12.9, 13.8 ± 3.7 g/kg for VS, TN, and TP respectively. Nutrient concentrations in the dry compost were higher and there was no significant difference found between litter compost and simple static pile compost. Since, dry compost only evaporates water from manure and it will not have same characteristics as of other compost i.e. humification and pathogen removal.

### Weight reduction during composting and storage process:

In order to calculate the weight loss during composting period, the amount of manure and compost should be investigated but the total amount of broiler manure could not be surveyed. The weight reduction rates for broiler and layer can be seen in Table 3. The field survey showed that the weight loss rate during the composting process was reduced by 86% and the weight loss rate calculated by the phosphorus concentration difference between manure and compost (ΔP = 0) decreased by 45%.

**Table 3:** Weight reduction cases (%) for broiler and laying hen according to composting method

Weight reduction cases	Broiler (% weight reduction)	Layer (% weight reduction)		
	Deep litter	Deep litter	Drying	Simple static pile
A (Farm Investigation)	86 ± 11	67 ± 28	64 ± 15	77 ± 5
B (ΔP = 0)	45 ± 18	54 ± 6	68 ± 12	49 ± 15
C (Dry basis)	–	–	86 ± 4	–

The weight loss rate of each laying hen’s composting method was obtained to calculate the NLCs. The weight loss rate was calculated as the ratio of manure to compost, but in case of litter, the manure could not be sampled on site because it was mixed with litter immediately as it was excreted. Therefore, the weight loss rate was calculated by citing the data of the ME only in the portion of manure generated in the litter house. During the composting process the weight loss rate decreased by 67% in litter compost, 64% in dry compost, and 77% in simple static pile compost. The weight reduction rate calculated by P concentration change (ΔP = 0) was found to be reduced by 54% in litter composting, 68% in dry composting, and 49% in simple static pile composting. The dry contents of the dry compost were calculated by using the water content difference between manure and compost. The weight loss rate of 86% was calculated by this method. Thompson et al. (1996) has reported a 67.2% weight reduction of raw manure of egg laying hen while 72-82% weight reduction after composting with pine shavings for 246 days using deep litter system [21].

### Nutrient loading coefficients:

Broiler and layer litter manure production was calculated using ME reports because it was not possible to investigate as manure was immediately mixed with litter after excretion. The NLCs of N and P for weight reduction scenario A were 0.32 and 0.23 for broilers and 0.79 and 0.67 for laying hens respectively (Table 4). The N and P reduction rates for broilers were 69% and 78% while for laying hens reduction rates were 21% and 33% respectively. For weight reduction scenario B, loading coefficient of N for broilers and laying hens was 1.22 and 1.27 respectively. The N for the scenario B was increased by 22% and 51%. The loading coefficient for P had zero loss as for both broiler and layer had a coefficient of 1.0. The NLCs of N for scenario B for broiler were about 4 times higher than the scenario A. This was because of the broiler shipment for slaughter after breeding period (35 days) and then water was added to the manure and litter mixture left in the farm for a month. The air in the farm house was completely shut off that made conditions anaerobic. In this case, the leachate was generated that was leached into the drainage or house floor. Therefore, it is estimated that the NLCs of the farm investigation are low because nutrients were escaped in the form of leachate. Conversely, NLCs obtained in Scenario B were higher because it included the nutrients of leachate as the load factor including the amount of nutrients exiting into leachate. The NLCs of N for layer were 2 times higher than the scenario B. The increased N contents are due to the prolonged breeding time of the laying hens compared to broilers. Unlike broilers the leaching loss was low despite using same composting method. Reason for comparatively low leaching in case of layers was due to no water addition and no accumulation period of a month.

**Table 4:** Nutrient loading coefficients according to manure production & weight reduction cases (Deep litter composting)

Weight reduction scenarios	Manure production cases^a^		
	Nutrients	Broiler	Layer
A (Farm Investigation)	VS	— ^b^	— ^b^
	N	0.32	0.79
	P	0.23	0.67
B (ΔP = 0)	VS	— ^b^	— ^b^
	N	1.22	1.51
	P	— ^c^	— ^c^

^a^Manure production reported from ME x nutrient concentrations in compost from farm samples

^b^Organic component of the ME is not to be compared referred to as BOD

^c^P coefficient is 1.0

In case of drying manure, the NLCs calculated from farm investigation for the [Case I x A], the VS, N, and P with the values of 1.44, 0.90, and 1.33 respectively (Table 5). For [Case I x B] the VS, N, and P were 1.36, 0.64, and 1.00 respectively. For [Case I x C] the values were 0.56, 0.33, and 0.47 respectively. The Case I and Case II had similar values. The reduction of N for case I was 10% for scenario A, 36% for scenario B, and 67% for scenario C while for P an increase of 33% was seen for scenario A and 0% for scenario B. The NLCs for P should not be more than 1.00 as NLC for farm investigation increased P due to drying therefore; NLCs were calculated on dry basis of the manure and compost (scenario C) that gave lowest NLCs among all scenarios (VS, 0.56; N, 0.33; P, 0.47) and are considered to be the most reliable. Because the composting method is drying in the house or drying by the machine, it seems that the nitrogen stored at high temperature and caused high reduction due to volatilization and since the decrease of nutrients seems to be large due to the occurrence of the leachate in the anaerobic state. In order to calculate the nutrient loading including the nutrients of the leachate, farm investigations scenarios 1 and 4 are considered to be appropriate for the reason of mixed feeds, instead of Scenario 2 and 5. For case III, N and P has shown an increase of 35% and 64% for scenario A while reduction of 20% and 0% for scenario B and 50% and 42% for scenario C respectively.

**Table 5:** Nutrient loading coefficients according to manure production & weight reduction cases (Dry composting)

Weight reduction scenarios	Manure production cases			
	Nutrients	Case I^a^	Case II	Case III
A (Farm Investigation)	VS	1.44	1.44	— ^b^
	N	0.90	0.90	1.35
	P	1.33	1.33	1.64
B (ΔP = 0)	VS	1.36	1.36	— ^b^
	N	0.64	0.64	0.80
	P	— ^c^	— ^c^	— ^b^
C (Dry basis)	VS	0.56	0.56	— ^b^
	N	0.33	0.33	0.50
	P	0.47	0.47	0.58

^a^Case I, manure production from questionnaire x nutrient concentrations in compost from farm sample; Case II, manure production reported by ME x nutrient concentrations in compost from farm sample; Case III, manure production reported from ME x nutrient concentrations in compost from ME

^b^Organic component of the ME is not to be compared referred to as BOD

^c^P coefficient is 1.0

In case of simple static pile, the NLCs calculated are summarized in the Table 6. The NLCs calculated from farm investigation for the [Case I x A], the VS, N, and P with the values of 0.52, 0.44, and 0.48 respectively. For [Case I x B] the VS, N, and *P* values were 1.2, 0.95, and 1.00 respectively. The manure Case I and Case II had more or less similar values. The reduction of N was 56% for scenario A and 5% for scenario B while for P, 52% and 0% reduction was seen for scenario A and scenario B. For case III that was calculated using ME reports for manure data, N and P has shown a reduction of 38% and 48% for scenario A, respectively while an increase of 19% and 0% for scenario B, respectively. The nitrogen reduction rate was lower than that of the dried compost, and VS and phosphorus were similar. Even lower than the scenario 2 and 4 that was because the laying manure was deposited for a certain period of time before mixing with sawdust and the nutrients were decreased due to leaching.

**Table 6:** Nutrient loading coefficients according to manure production & weight reduction cases (Simple static pile composting)

Weight reduction scenarios	Manure production cases			
	Nutrients	Case I^a^	Case II	Case III
A (Farm Investigation)	VS	0.52	0.51	— ^b^
	N	0.44	0.43	0.62
	P	0.48	0.47	0.52
B (ΔP = 0)	VS	1.20	1.20	— ^b^
	N	0.95	0.95	1.19
	P	— ^c^	— ^c^	— ^c^

^a^Case I, manure production from questionnaire x nutrient concentrations in compost from farm sample; Case II, manure production reported by ME x nutrient concentrations in compost from farm sample; Case III, manure production reported from ME x nutrient concentrations in compost from ME

^b^ = Organic component of the ME is not to be compared referred to as BOD

^c^ = P coefficient is 1.0

The NLCs for VS were higher than 1.0 due to the addition of bulking material except for simple static pile where 48% VS was lost may be due to long period of composting. In case of ME values (case III) the VS were reported as BOD so it was not considered. Maximum N reduction was seen for broilers (69%) and for Layer in the range of 10-56% in farm investigation. Morand et al. 2005 reported a decrease of 61-74% of initial N in poultry manure composting while Ghaly et al. 2013 has reported a 44-55% N loss during poultry manure drying [10, 22]. The loss of phosphorus for broiler was78% and for layer it was in the range of 33-52% in farm investigation. Phosphorus is usually lost either by leaching or run off during composting. Vadas et al. (2004) has reported that the P contents of poultry manure decreased by 50% before composting due to dilution with low P composting materials [23]. Theoretically, the amount of P cannot be changed as the VS and N are reduced due to the oxidation of the materials. The P amount can be the indicator for weight loss as P is not removed from compost [7]. Uptake of P by microbes occurs but it remains in the system (as poly-PO_4_) hence weight reduction rates for P in scenario B were 1.0. The NLCs were largely influenced by the weight reduction due to composting method and storing period instead of manure production amount [7].

## Conclusions

Overestimation of the nutrients for the agricultural land has surpassed cropland demand. So, to stop eutrophication and over nourishment of the land it is feasible to calculate nutrient budget. In this study, the nutrients in manure from farm investigation were lost due to microbial action, storing, and drying during composting period. During farm investigation of broiler and layer litter, there was a 68 and 21% N loss whereas 77 and 33% P loss was found, respectively. In case of layer composting, a loss of 10-56% N and a 52% P loss was observed. Drying manure increased the P concentrations therefore NLCs calculated using dry basis that showed quite higher reductions (67% N; 53% P). In all cases the nutrient losses calculated were higher than those of ME. This suggests that the mass balance of poultry manure nutrients applied over cropland is overestimated and needs to be corrected and updated. To stop over nutrient application the proper national nutrient management program should be created.

## Abbreviations

BOD: Biological oxygen demand
C: Carbon
°C: Degree Celsius
CH_4_: Methane
Cl: Chlorine
CO_2_: Carbon dioxide
d: Day
DOC: Dissolved organic carbon
Eq.: Equation
g: Gram
h: Hour
HCO_3_: Bicarbonate
K: Potassium
kg: Kilogram
kt: kilo tons
L: Litter
M: Million
m^3^: Cubic meter
ME: Ministry of Environment
mg: Milligram
MMB: Mixture of manure and bulking material
N: Nitrogen
N_2_O: Nitrous oxide
Na: Sodium
NH_3_: Ammonia
NLC: Nutrient loading coefficient
OECD: The Organization for Economic Co-operation and Development
P: Phosphorus
PO_4_: Phosphate
S: Sulfur
TN: Total nitrogen
TP: Total phosphorus
TS: Total solids
VS: Volatile solids
ΔP: Changes in phosphorus concentrations
